# 4-Hy­droxy­pyridinium-3-sulfonate

**DOI:** 10.1107/S1600536810049603

**Published:** 2010-12-04

**Authors:** Zhi-Biao Zhu, Shan Gao, Seik Weng Ng

**Affiliations:** a College of Chemistry and Materials Science, Heilongjiang University, Harbin 150080, People’s Republic of China; b Department of Chemistry, University of Malaya, 50603 Kuala Lumpur, Malaysia

## Abstract

The reaction of 4-hy­droxy­pyridine and oleum produces 4-hy­droxy­pyridinium-3-sulfonate, C_5_H_5_NO_4_S, which shows delocalized bonds in the six-membered ring. In the crystal, adjacent zwitterions are linked by N—H⋯O and O—H⋯O hydrogen bonds into a layer motif. The crystal studied was a racemic twin.

## Related literature

A previous synthesis yielded hydro­nium 4-oxo-1,4-dihydro­pyridine-3-sulfonate dihydrate; see: Zhu *et al.* (2009 ▶).
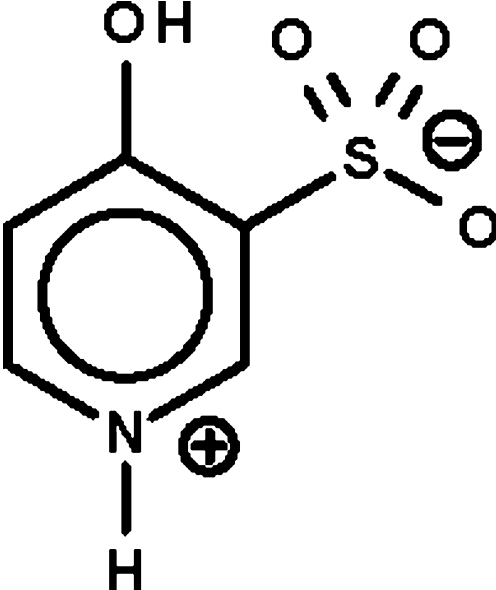


## Experimental

### 

#### Crystal data

C_5_H_5_NO_4_S
*M* *_r_* = 175.16
Orthorhombic, 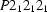

*a* = 6.7980 (2) Å
*b* = 8.7618 (3) Å
*c* = 10.6797 (3) Å
*V* = 636.11 (3) Å^3^
*Z* = 4
Mo *K*α radiation
μ = 0.47 mm^−1^
*T* = 293 K
0.28 × 0.23 × 0.17 mm

#### Data collection

Rigaku R-AXIS RAPID diffractometer
Absorption correction: multi-scan (*ABSCOR*; Higashi, 1995 ▶) *T*_min_ = 0.880, *T*_max_ = 0.925
6216 measured reflections
1449 independent reflections
1403 reflections with *I* > 2σ(*I*)
*R*_int_ = 0.017

#### Refinement

*R*[*F* ^2^ > 2σ(*F* ^2^)] = 0.024
*wR*(*F* ^2^) = 0.068
*S* = 1.09
1449 reflections
121 parameters
5 restraints
All H-atom parameters refined
Δρ_max_ = 0.25 e Å^−3^
Δρ_min_ = −0.23 e Å^−3^
Absolute structure: Flack (1983 ▶), 785 Friedel pairs
Flack parameter: 0.31 (8)

### 

Data collection: *RAPID-AUTO* (Rigaku, 1998 ▶); cell refinement: *RAPID-AUTO*; data reduction: *CrystalStructure* (Rigaku/MSC, 2002 ▶); program(s) used to solve structure: *SHELXS97* (Sheldrick, 2008 ▶); program(s) used to refine structure: *SHELXL97* (Sheldrick, 2008 ▶); molecular graphics: *X-SEED* (Barbour, 2001 ▶); software used to prepare material for publication: *publCIF* (Westrip, 2010 ▶).

## Supplementary Material

Crystal structure: contains datablocks global, I. DOI: 10.1107/S1600536810049603/im2249sup1.cif

Structure factors: contains datablocks I. DOI: 10.1107/S1600536810049603/im2249Isup2.hkl

Additional supplementary materials:  crystallographic information; 3D view; checkCIF report

## Figures and Tables

**Table 1:** Hydrogen-bond geometry (Å, °)

*D*—H⋯*A*	*D*—H	H⋯*A*	*D*⋯*A*	*D*—H⋯*A*
O1—H1*O*⋯O2^i^	0.83 (1)	1.76 (1)	2.581 (2)	166 (3)
N1—H1n⋯O3^ii^	0.87 (1)	1.91 (1)	2.762 (2)	166 (2)

Symmetry codes: (i) 
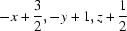
; (ii) 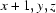.

**Fig. 1.**
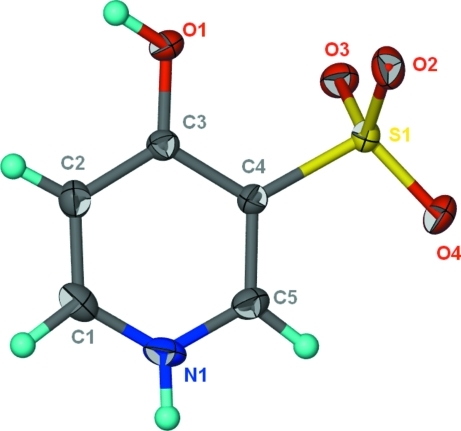
Thermal ellipsoid plot (Barbour, 2001) of C5H5NO4S at the 50% probability level; hydrogen atoms are drawn as spheres of arbitrary radius.

**Fig. 2.**
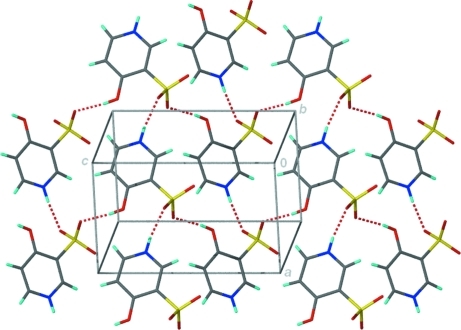
Hydrogen-bonded layer structure.

## supplementary crystallographic information

### Comment

A previous reaction of 4-hydroxpyridine and oleum gave the salt, hydronium 4-oxo-1,4-dihydropyridine-3-sulfonate dihydrate (Zhu *et al.*, 2009). Repeating this synthesis instead produced the zwitterionic title compound (Scheme I, Fig. 1). The bonds in the ring are delocalized bonds. Adjacent zwitterions are linked by N–H···O and O–H···O hydrogen bonds into a layer motif (Fig. 2).

### Experimental

4-Hydroxypyridine (10 mmol) was dissolved in 20% oleum (10 ml). The solution was heated to 393 K for 4 days. After it was cooled to room temperature, the excess oleum was decanted. Recrystallization of the solid from ethanol gave colorless crystals.

### Refinement

Carbon-bound H atoms were refind with a C–H 0.95±0.01 Å restraint. The amino and hydroxy H atoms were located in a difference Fourier map, and were refined with distance restraints of O–H 0.84±0.01 Å and N–H 0.88±0.01 Å. All temperature factors were refined.

### Crystal data

**Table:**

C_5_H_5_NO_4_S	*F*(000) = 360
*M**_r_* = 175.16	*D*_x_ = 1.829 Mg m^−^^3^
Orthorhombic, *P*2_1_2_1_2_1_	Mo *K*α radiation, λ = 0.71073 Å
Hall symbol: P 2ac 2ab	Cell parameters from 6017 reflections
*a* = 6.7980 (2) Å	θ = 3.0–27.4°
*b* = 8.7618 (3) Å	µ = 0.47 mm^−^^1^
*c* = 10.6797 (3) Å	*T* = 293 K
*V* = 636.11 (3) Å^3^	Prism, colorless
*Z* = 4	0.28 × 0.23 × 0.17 mm

### Data collection

**Table:**

Rigaku R-AXIS RAPID diffractometer	1449 independent reflections
Radiation source: fine-focus sealed tube	1403 reflections with *I* > 2σ(*I*)
graphite	*R*_int_ = 0.017
Detector resolution: 10.000 pixels mm^-1^	θ_max_ = 27.4°, θ_min_ = 3.0°
ω scans	*h* = −8→7
Absorption correction: multi-scan (*ABSCOR*; Higashi, 1995)	*k* = −11→11
*T*_min_ = 0.880, *T*_max_ = 0.925	*l* = −13→13
6216 measured reflections	

### Refinement

**Table:**

Refinement on *F*^2^	Secondary atom site location: difference Fourier map
Least-squares matrix: full	Hydrogen site location: inferred from neighbouring sites
*R*[*F*^2^ > 2σ(*F*^2^)] = 0.024	All H-atom parameters refined
*wR*(*F*^2^) = 0.068	*w* = 1/[σ^2^(*F*_o_^2^) + (0.0488*P*)^2^ + 0.0899*P*] where *P* = (*F*_o_^2^ + 2*F*_c_^2^)/3
*S* = 1.09	(Δ/σ)_max_ = 0.001
1449 reflections	Δρ_max_ = 0.25 e Å^−^^3^
121 parameters	Δρ_min_ = −0.23 e Å^−^^3^
5 restraints	Absolute structure: Flack (1983), 785 Friedel pairs
Primary atom site location: structure-invariant direct methods	Flack parameter: 0.31 (8)

### Fractional atomic coordinates and isotropic or equivalent isotropic displacement parameters (Å^2^)

**Table:**

	*x*	*y*	*z*	*U*_iso_*/*U*_eq_	
S1	0.84628 (5)	0.31587 (4)	0.15713 (3)	0.02340 (12)	
O1	0.81623 (18)	0.44053 (16)	0.41523 (12)	0.0304 (3)	
O2	0.7512 (2)	0.46465 (15)	0.13994 (12)	0.0348 (3)	
O3	0.71610 (16)	0.20834 (15)	0.21966 (11)	0.0307 (3)	
O4	0.9407 (2)	0.25936 (17)	0.04580 (11)	0.0344 (3)	
N1	1.37517 (19)	0.33180 (18)	0.31720 (15)	0.0310 (3)	
C1	1.3417 (3)	0.3866 (2)	0.43239 (17)	0.0317 (4)	
C2	1.1563 (3)	0.42245 (19)	0.47075 (15)	0.0282 (3)	
C3	0.9993 (2)	0.40609 (18)	0.38677 (14)	0.0224 (3)	
C4	1.0389 (2)	0.34817 (17)	0.26608 (14)	0.0216 (3)	
C5	1.2292 (2)	0.3108 (2)	0.23489 (15)	0.0274 (3)	
H1O	0.794 (5)	0.485 (3)	0.4827 (16)	0.064 (9)*	
H1N	1.4924 (18)	0.306 (3)	0.2930 (19)	0.036 (6)*	
H1	1.456 (2)	0.394 (3)	0.4826 (19)	0.038 (6)*	
H2	1.138 (3)	0.463 (2)	0.5519 (11)	0.021 (4)*	
H5	1.267 (3)	0.263 (3)	0.1575 (14)	0.042 (6)*	

### Atomic displacement parameters (Å^2^)

**Table:**

	*U*^11^	*U*^22^	*U*^33^	*U*^12^	*U*^13^	*U*^23^
S1	0.02372 (18)	0.02877 (19)	0.01770 (17)	0.00307 (16)	−0.00047 (14)	−0.00191 (14)
O1	0.0236 (6)	0.0426 (7)	0.0249 (6)	0.0052 (5)	0.0026 (5)	−0.0082 (5)
O2	0.0429 (7)	0.0352 (7)	0.0262 (6)	0.0118 (5)	−0.0080 (6)	0.0003 (5)
O3	0.0259 (5)	0.0354 (6)	0.0308 (6)	−0.0021 (5)	0.0030 (5)	−0.0030 (5)
O4	0.0361 (6)	0.0460 (7)	0.0212 (5)	0.0012 (6)	0.0036 (5)	−0.0086 (5)
N1	0.0174 (6)	0.0363 (8)	0.0393 (8)	0.0029 (6)	0.0028 (5)	0.0047 (6)
C1	0.0258 (8)	0.0326 (8)	0.0368 (9)	−0.0014 (7)	−0.0071 (8)	0.0052 (7)
C2	0.0298 (8)	0.0321 (7)	0.0227 (7)	−0.0004 (8)	−0.0030 (7)	0.0001 (6)
C3	0.0211 (7)	0.0237 (7)	0.0224 (7)	0.0004 (6)	0.0014 (6)	0.0017 (5)
C4	0.0210 (7)	0.0249 (7)	0.0189 (6)	0.0016 (5)	0.0009 (5)	0.0011 (6)
C5	0.0253 (7)	0.0296 (7)	0.0273 (7)	0.0022 (7)	0.0063 (6)	0.0018 (7)

### Geometric parameters (Å, °)

**Table:**

S1—O4	1.4390 (12)	N1—H1N	0.868 (10)
S1—O3	1.4549 (12)	C1—C2	1.362 (3)
S1—O2	1.4666 (13)	C1—H1	0.947 (10)
S1—C4	1.7747 (15)	C2—C3	1.402 (2)
O1—C3	1.316 (2)	C2—H2	0.943 (9)
O1—H1O	0.834 (10)	C3—C4	1.411 (2)
N1—C5	1.338 (2)	C4—C5	1.375 (2)
N1—C1	1.340 (2)	C5—H5	0.961 (10)
			
O4—S1—O3	115.32 (8)	C1—C2—C3	119.25 (16)
O4—S1—O2	113.52 (8)	C1—C2—H2	119.0 (13)
O3—S1—O2	111.40 (8)	C3—C2—H2	121.6 (13)
O4—S1—C4	105.51 (7)	O1—C3—C2	123.29 (15)
O3—S1—C4	104.55 (7)	O1—C3—C4	118.31 (14)
O2—S1—C4	105.41 (7)	C2—C3—C4	118.40 (15)
C3—O1—H1O	118 (2)	C5—C4—C3	119.11 (14)
C5—N1—C1	121.76 (14)	C5—C4—S1	119.83 (12)
C5—N1—H1N	116.7 (15)	C3—C4—S1	121.01 (11)
C1—N1—H1N	121.5 (15)	N1—C5—C4	120.37 (15)
N1—C1—C2	121.07 (16)	N1—C5—H5	115.2 (14)
N1—C1—H1	113.9 (15)	C4—C5—H5	124.4 (14)
C2—C1—H1	125.0 (15)		
			
C5—N1—C1—C2	0.4 (3)	O3—S1—C4—C5	118.79 (15)
N1—C1—C2—C3	−2.0 (3)	O2—S1—C4—C5	−123.66 (15)
C1—C2—C3—O1	−178.80 (16)	O4—S1—C4—C3	179.33 (13)
C1—C2—C3—C4	1.9 (2)	O3—S1—C4—C3	−58.62 (15)
O1—C3—C4—C5	−179.69 (15)	O2—S1—C4—C3	58.93 (15)
C2—C3—C4—C5	−0.4 (2)	C1—N1—C5—C4	1.2 (3)
O1—C3—C4—S1	−2.3 (2)	C3—C4—C5—N1	−1.2 (3)
C2—C3—C4—S1	177.04 (12)	S1—C4—C5—N1	−178.64 (12)
O4—S1—C4—C5	−3.26 (17)		

### Hydrogen-bond geometry (Å, °)

**Table:**

*D*—H···*A*	*D*—H	H···*A*	*D*···*A*	*D*—H···*A*
O1—H1O···O2^i^	0.83 (1)	1.76 (1)	2.581 (2)	166 (3)
N1—H1n···O3^ii^	0.87 (1)	1.91 (1)	2.762 (2)	166 (2)

Symmetry codes: (i) −*x*+3/2, −*y*+1, *z*+1/2; (ii) *x*+1, *y*, *z*.
